# Multi-Scale Frequency-Aware Transformer for Pipeline Leak Detection Using Acoustic Signals

**DOI:** 10.3390/s25206390

**Published:** 2025-10-16

**Authors:** Menghan Chen, Yuchen Lu, Wangyu Wu, Yanchen Ye, Bingcai Wei, Yao Ni

**Affiliations:** 1 School of Integrated Circuit Engineering, Guangdong University of Technology, Guangzhou 510006, China; cmmh@hrbeu.edu.cn (M.C.); luyuchen@hrbeu.edu.cn (Y.L.); 2108550023037@stu.bucea.edu.cn (Y.Y.); 2 Yantai Research Institute, Harbin Engineering University, Yantai 264005, China; 3 School of Computer Science, University of Liverpool, Liverpool L69 7ZX, UK; wangyu.wu@liverpool.ac.uk; 4 School of Mechanical, Electrical and Vehicle Engineering, Beijing University of Civil Engineering and Architecture, Beijing 100044, China; 5 School of Computer Science, Wuhan University, Wuhan 430072, China; weibc97@whu.edu.cn

**Keywords:** leak detection, acoustic measurement, MSFAT, noise filtering, frequency attention

## Abstract

Pipeline leak detection through acoustic signal measurement faces critical challenges, including insufficient utilization of time-frequency domain features, poor adaptability to noisy environments, and inadequate exploitation of frequency-domain prior knowledge in existing deep learning approaches. This paper proposes a Multi-Scale Frequency-Aware Transformer (MSFAT) architecture that integrates measurement-based acoustic signal analysis with artificial intelligence techniques. The MSFAT framework consists of four core components: a frequency-aware embedding layer that achieves joint representation learning of time-frequency dual-domain features through parallel temporal convolution and frequency transformation, a multi-head frequency attention mechanism that dynamically adjusts attention weights based on spectral distribution using frequency features as modulation signals, an adaptive noise filtering module that integrates noise detection, signal enhancement, and adaptive fusion functions through end-to-end joint optimization, and a multi-scale feature aggregation mechanism that extracts discriminative global representations through complementary pooling strategies. The proposed method addresses the fundamental limitations of traditional measurement-based detection systems by incorporating domain-specific prior knowledge into neural network architecture design. Experimental validation demonstrates that MSFAT achieves 97.2% accuracy and an F1-score, representing improvements of 10.5% and 10.9%, respectively, compared to standard Transformer approaches. The model maintains robust detection performance across signal-to-noise ratio conditions ranging from 5 to 30 dB, demonstrating superior adaptability to complex industrial measurement environments. Ablation studies confirm the effectiveness of each innovative module, with frequency-aware mechanisms contributing most significantly to the enhanced measurement precision and reliability in pipeline leak detection applications.

## 1. Introduction

As a clean and efficient energy source, the widespread application of natural gas in industrial, commercial, and residential sectors has made the safety and reliability of pipeline transportation systems a critical guarantee for energy security and social stability [1,2,3,4]. Leakage accidents caused by factors such as pipeline aging, corrosion, and third-party damage not only result in enormous economic losses but may also trigger serious safety incidents such as explosions [5,6,7]. According to statistics, pipeline leakage is the primary failure mode of natural gas transmission and distribution systems, accounting for approximately 60–70% of all pipeline accidents [8,9]. Therefore, developing high-precision and robust pipeline leakage detection technologies is of significant importance for ensuring energy transportation safety and reducing economic losses [10,11,12].

Among various technical approaches for pipeline leakage detection, metrological detection methods based on acoustic signals have become the primary technological pathway widely adopted by industry due to their unique advantages of long propagation distance, fast response speed, and non-contact measurement [13,14,15]. Compared to other detection methods using pressure signals, vibration signals, and other approaches, acoustic signals possess significant metrological advantages: First, the frequency response range of acoustic sensors extends from infrasonic to ultrasonic waves, enabling precise measurement of fault characteristic information across the full frequency spectrum [16,17,18]. Second, acoustic detection features non-destructive characteristics, allowing continuous online monitoring without affecting normal pipeline operation [19,20]. Finally, acoustic signals possess the physical property of penetrating obstacles for long-distance propagation, making them particularly suitable for distributed metrological applications in complex industrial environments [21,22,23].

Traditional acoustic signal processing methods primarily rely on manual feature extraction techniques such as wavelet transform [16], empirical mode decomposition [24], and variational mode decomposition combined with shallow classifiers, including support vector machines, artificial neural networks, and random forests [25,26,27,28,29,30,31]. These methods typically employ enhanced local mean mode decomposition algorithms for noise suppression or use numerical integrators to amplify low-frequency signal features [32,33,34,35,36]. Although they can achieve leakage detection to a certain extent, they often require extensive professional knowledge and complex parameter design, and shallow architectures struggle to handle complex nonlinear features in realistic environments. Furthermore, traditional methods typically adopt a two-stage processing workflow: feature extraction and fault classification, where this separated design limits the optimization space of the overall system [37,38,39].

In recent years, artificial intelligence technologies, particularly deep learning, have demonstrated powerful end-to-end learning capabilities in the field of pipeline leakage detection, providing a new technological paradigm for metrology-based fault diagnosis [40,41,42]. Intelligent architectures such as convolutional neural networks, autoencoders, and recurrent neural networks have achieved significant progress in acoustic signal feature representation and pattern recognition through their advanced network structures and powerful nonlinear learning capabilities [43,44,45,46]. Research studies on architectures integrating spectral enhancement with convolutional neural networks and methods combining frequency-domain vector denoising with time-domain correlation function enhancement demonstrate that deep learning models possess obvious advantages over traditional metrological methods in processing large-scale data and complex pattern recognition, achieving a technological leap from traditional metrological analysis to intelligent metrological diagnosis [47,48,49,50,51].

However, existing deep learning methods [52,53,54,55,56] still face three key challenges when processing acoustic signals for pipeline leakage. First, the insufficient utilization of time-frequency domain metrological features. Traditional methods only focus on metrological analysis of single features in either the time domain or frequency domain, failing to fully utilize the inherent time-frequency dual-domain metrological characteristics of acoustic signals, resulting in incomplete feature representation. Acoustic signals are essentially time-varying frequency-domain signals [57], and their leakage features are often distributed within specific time-frequency regions, making single-domain metrological processing approaches difficult to capture such complex time-frequency coupling relationships. Second, the metrological accuracy assurance problem under noisy environments. Complex and variable background noise in industrial environments, such as those with distinct hydrodynamic noise characteristics [58], severely affects the precise measurement of weak leakage signals, while existing pre-processing denoising methods adopt fixed filtering strategies that are difficult to adapt to dynamically changing noise environments [59]. Pipeline leakage signals are affected by factors such as pipeline materials and length during propagation, with high-frequency components particularly susceptible to attenuation during signal propagation, and background noise interference further impeding the precise measurement of effective leakage signal features. Finally, the insufficient integration of frequency-domain prior knowledge with artificial intelligence models. Standard deep learning architectures lack effective utilization of frequency-domain metrological prior knowledge of acoustic signals, and attention mechanisms cannot dynamically adjust weight allocation according to spectral distribution. Although traditional multi-head self-attention mechanisms can model dependencies within sequences, they lack effective utilization of frequency-domain metrological prior knowledge and struggle to accurately identify leakage-related spectral features.

Addressing the aforementioned challenges, the Multi-Scale Frequency-Aware Transformer (MSFAT) architecture provides a new technological paradigm for pipeline leakage detection through deep integration of the physical properties of acoustic signals with neural network design. MSFAT adopts an end-to-end deep learning framework that deeply embeds the physical mechanisms of acoustic signals into neural network architecture design. The frequency-aware embedding layer breaks through the limitations of traditional methods that only process single-domain features, achieving time-frequency feature fusion through parallel time-domain convolution and frequency-domain transformation, providing a rich representational foundation for subsequent processing. The multi-head frequency attention mechanism utilizes spectral features as modulation signals, dynamically adjusting attention weights according to the spectral distribution of input signals, effectively utilizing frequency-domain prior knowledge to guide feature learning. The adaptive noise filtering module integrates noise detection, signal enhancement, and adaptive fusion functions, achieving adaptive processing strategies for different noise levels through end-to-end joint optimization. The multi-scale feature aggregation mechanism extracts discriminative global representations through complementary pooling strategies, further enhancing the overall performance of the model.

Experimental validation demonstrates that MSFAT achieves 97.2% in both accuracy and F1-score, representing improvements of 10.5% and 10.9%, respectively, compared to the standard Transformer, exhibiting excellent robustness within the 5–30 dB signal-to-noise ratio range. Ablation experiments further confirm the effectiveness of each innovative module, with the frequency-aware mechanism contributing most significantly. The main contributions of this research include the following:

1. Proposing a frequency-aware embedding layer that achieves joint representation learning of time-frequency dual-domain features through parallel time-domain convolution and frequency-domain transformation, breaking through the limitations of traditional methods’ single-domain feature processing.
2. Designing a multi-head frequency attention mechanism that utilizes spectral features as modulation signals, dynamically adjusting attention weights based on spectral distribution to achieve effective utilization of frequency-domain prior knowledge.
3. Constructing an adaptive noise filtering module that integrates noise detection, signal enhancement, and adaptive fusion functions, achieving adaptive noise suppression under wide-range signal-to-noise ratio conditions through end-to-end joint optimization.

## 2. Methodology

### 2.1. MSFAT Overview

**Figure 1:**
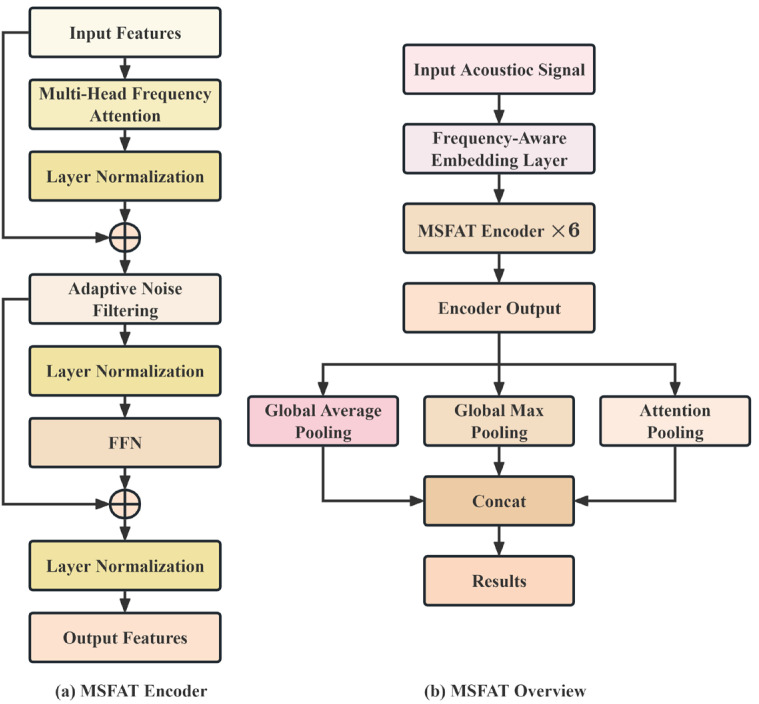
MSFAT architecture schematic diagram.

The Multi-Scale Frequency-Aware Transformer (MSFAT) architecture proposed in this paper is specifically designed to address the acoustic signal classification problem in pipeline leak detection. As illustrated in Figure 1, MSFAT employs an end-to-end deep learning framework, primarily composed of the following four core modules:

- Frequency-aware embedding layer for learning a joint representation of features in both time and frequency domains.
- Multi-head frequency attention mechanism, which dynamically adjusts attention weight distribution based on spectral characteristics.
- Adaptive noise filtering module for end-to-end noise suppression across a wide range of signal-to-noise ratio (SNR) variations.
- Multi-scale feature aggregation mechanism, which extracts discriminative global representations through complementary pooling strategies.

Compared to traditional signal processing methods based on handcrafted features and standard deep learning architectures, the primary innovation of MSFAT lies in deeply integrating the physical characteristics of acoustic signals into the neural network design. Specifically, the frequency-aware embedding layer overcomes the limitation of traditional methods that focus solely on single-domain features, the multi-head frequency attention mechanism resolves the issue of standard Transformers being unable to effectively utilize prior frequency-domain knowledge, and the adaptive noise filtering module achieves superior end-to-end joint optimization compared to pre-processing denoising methods.

### 2.2. Problem Formulation

Let the input acoustic signal be $x\in {\mathbb{R}}^{T}$, where enotes the layer index represents the number of sampling points. The pipeline leak detection task can be formalized as a three-class classification problem:

(1) $$\hat{y}=\arg\max_{c\in \{0,1,2\}}P(y=c|x)$$

where c=0,1,2 correspond to no-leakage, hole-leakage, and valve-leakage states, respectively.

The MSFAT model learns a mapping function $f_{\theta }:{\mathbb{R}}^{T}\to {\mathbb{R}}^{3}$, where θ denotes the model parameters, with the objective of achieving high-accuracy classification in complex acoustic environments with an SNR range of 5–30 dB.

### 2.3. Frequency-Aware Embedding Layer

Traditional Transformer embedding layers typically process inputs from a single domain, failing to fully exploit the inherent time-frequency characteristics of acoustic signals. To address this issue, this paper designs a frequency-aware embedding layer that achieves joint time-frequency feature representation learning through parallel time-domain convolutions and frequency-domain transformations.

Given an input signal $x\in {\mathbb{R}}^{T}$, multi-scale convolutional strategies are employed for time-domain feature extraction. The multi-scale time-domain features are computed as follows:

(2) $$h_{i}^{\left(t\right)}=\mathrm{ReLU}(x*W_{i}^{\left(t\right)}),\;i\in \{1,2,3,4\}$$

where * denotes the one-dimensional convolution operation, $W_{i}^{\left(t\right)}\in {\mathbb{R}}^{k_{i}\times d/4}$ is the convolution weight matrix for the i-th kernel with its size $k_{i}$ chosen from {3,7,15,31}, and d is the embedding dimension.

Features from different scales are concatenated to form a complete time-domain representation:

(3) $$H^{\left(t\right)}=\mathrm{Concat}[h_{1}^{\left(t\right)},h_{2}^{\left(t\right)},h_{3}^{\left(t\right)},h_{4}^{\left(t\right)}]\in {\mathbb{R}}^{T\times d}$$

In parallel, frequency-domain features are obtained via the real-valued fast Fourier transform (RFFT). The frequency-domain amplitude spectrum is defined as follows:

(4) $$X_{f}=|\mathrm{RFFT}(x)|\in {\mathbb{R}}^{F}$$

where $F=\left[T/2\right]+1$ is the frequency-domain dimension. To adaptively enhance leak-related frequency bands, a frequency-domain enhancement network is introduced as follows:

(5) $$f_{base}=W_{fae,f}X_{f}+b_{fae,f}$$

(6) $$w_{enhance}=\sigma (W_{fae,e2}\mathrm{ReLU}(W_{fae,e1}f_{base}))$$

where $W_{fae,f}\in {\mathbb{R}}^{d\times F}$ and $b_{fae,f}\in {\mathbb{R}}^{d}$ are the weight matrix and bias for base frequency feature extraction, while $W_{fae,e1}\in {\mathbb{R}}^{d\times d}$ and $W_{fae,e2}\in {\mathbb{R}}^{d\times d}$ are the weight matrices for the enhancement network, respectively. The function σ(⋅) is the sigmoid activation.

The enhanced frequency-domain features are extended to the sequence length through a time-dimension replication operation:

(7) $$H^{\left(f\right)}=1_{T}⊗(f_{base}⊙w_{enhance})\in {\mathbb{R}}^{T\times d}$$

where $1_{T}\in {\mathbb{R}}^{T}$ is a vector of all ones, ⊗ denotes the outer product operation, and ⊙ denotes element-wise multiplication.

Finally, time-frequency features are adaptively fused, and positional information is injected using a learnable weight parameter:

(8) $$H=\sigma (\alpha )H^{\left(t\right)}+(1-\sigma (\alpha ))H^{\left(f\right)}+PE(H^{\left(t\right)})$$

where α∈ℝ is a learnable scalar fusion parameter and $PE(\cdot ):{\mathbb{R}}^{T\times d}\to {\mathbb{R}}^{T\times d}$ represents the sinusoidal positional encoding function. The resulting feature matrix H serves as the final output of the embedding layer.

**Figure 2:**
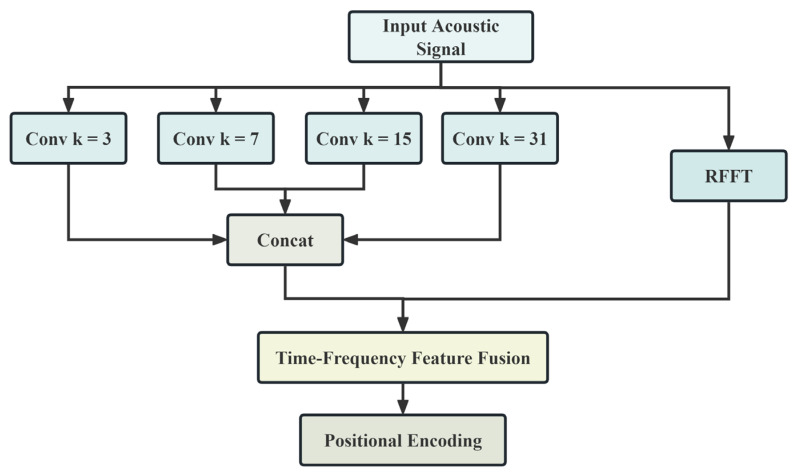
The architecture diagram of the frequency-aware embedding layer.

The architecture of the frequency-aware embedding layer is shown in Figure 2.

### 2.4. Multi-Head Frequency Attention

Standard multi-head self-attention mechanisms compute attention weights based on content similarity, a design based on semantic relevance that performs excellently in natural language processing tasks. However, for acoustic signal analysis, this design has obvious limitations: it cannot effectively utilize prior frequency-domain knowledge to guide attention allocation. To address this problem, this paper proposes a multi-head frequency attention mechanism, which uses spectral features as an additional modulating signal to achieve frequency-aware attention computation.

Given the embedded features $H\in {\mathbb{R}}^{T\times d}$, we first compute their mean along the feature dimension to obtain a time series representation and then extract spectral features via RFFT:

(9) $$X_{spec}=|\mathrm{RFFT}(\frac{1}{d}\sum_{i=1}^{d}H_{:,i})|\in {\mathbb{R}}^{F}$$

where $H_{:,i}$ denotes the time series of the *i*-th feature dimension. The spectral features $X_{spec}$ are then mapped through a multi-layer perceptron (MLP) to obtain weight allocations for h attention heads:

(10) $$w_{freq}=\mathrm{Softmax}(W_{mhfa,3}^{\left(f\right)}\mathrm{ReLU}(W_{mhfa,2}^{\left(f\right)}\mathrm{ReLU}(W_{mhfa,1}^{\left(f\right)}X_{spec})))\in {\mathbb{R}}^{h}$$

where $W_{mhfa,1}^{\left(f\right)}\in {\mathbb{R}}^{d\times F}$, $W_{mhfa,2}^{\left(f\right)}\in {\mathbb{R}}^{d/2\times d}$, and $W_{mhfa,3}^{\left(f\right)}\in {\mathbb{R}}^{h\times d/2}$ are the weight matrices of the MLP and $w_{freq}$ is the resulting vector of frequency-aware weights for the attention heads.

For the *j*-th attention head, the query, key, and value matrices are computed following the standard Transformer architecture:

(11) $$Q_{j}=HW_{Q}^{\left(j\right)},\;K_{j}=HW_{K}^{\left(j\right)},\;V_{j}=HW_{V}^{\left(j\right)}$$

where $W_{Q}^{\left(j\right)},W_{K}^{\left(j\right)},W_{V}^{\left(j\right)}\in {\mathbb{R}}^{d\times d_{k}}$ are the learnable projection matrices for the *j*-th head and $d_{k}=d/h$ is the feature dimension for each head. Frequency-aware attention performs global modulation of the standard attention scores using frequency weights:

(12) $$A_{j}=w_{freq,j}\cdot \mathrm{Softmax}\left(\frac{Q_{j}K_{j}^{T}}{\sqrt{d_{k}}}\right)\in {\mathbb{R}}^{T\times T}$$

where $w_{freq,j}$ is the *j*-th scalar component of the vector $w_{freq,j}$. This design enables different attention heads to adaptively focus on corresponding frequency band features based on the spectral distribution of the input signal. The multi-head outputs are obtained by concatenation and linear transformation:

(13) $$H_{\mathrm{mhfa}}=\mathrm{Concat}[A_{1}V_{1},\ldots ,A_{h}V_{h}]W_{O}$$

where $W_{O}\in {\mathbb{R}}^{d\times d}$ is the output projection matrix and $H_{\mathrm{mhfa}}$ is the final output of the multi-head frequency attention module. Compared to standard multi-head attention, this mechanism can better handle frequency-heterogeneous features in pipeline leak detection.

**Figure 3:**
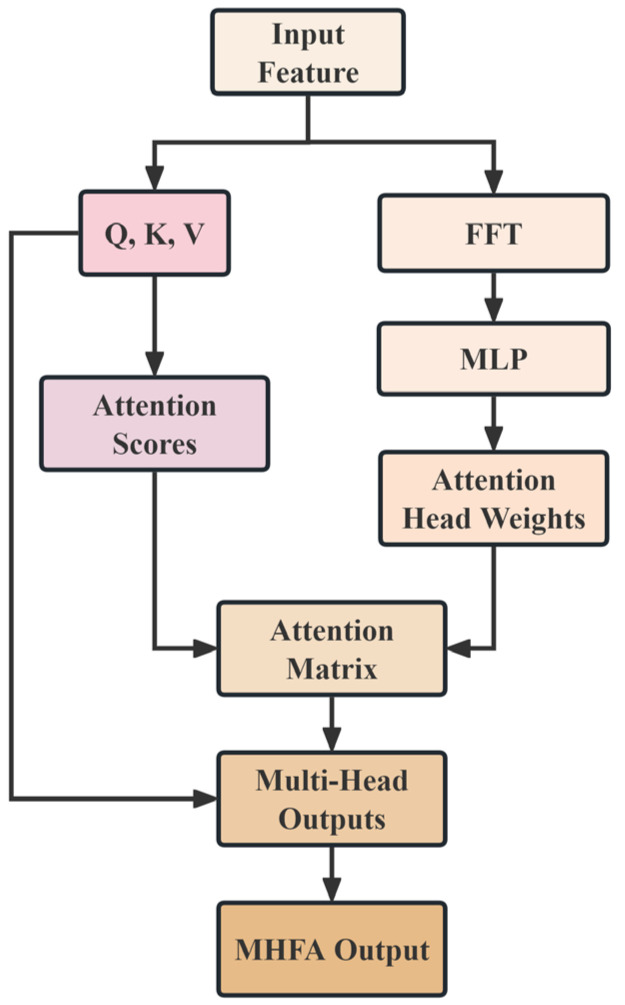
The architecture diagram of the multi-head frequency attention.

The architecture of the multi-head frequency attention is shown in Figure 3.

### 2.5. Adaptive Noise Filter

Pipeline leak detection in industrial environments faces complex noise interference, and traditional pre-processing denoising methods often employ fixed filtering strategies, making it difficult to adapt to dynamically changing noise environments. In contrast, the adaptive noise filtering (ANF) module designed in this paper integrates noise suppression directly into the deep network, enabling end-to-end joint optimization. The core idea of this module is to learn three sub-functions—noise detection, signal enhancement, and adaptive fusion—to enable MSFAT to maintain stable detection performance under a wide range of SNR conditions.

Given input features $H\in {\mathbb{R}}^{T\times d}$, the noise level estimation network predicts the local noise intensity at each time step:

(14) $$n_{t}=\sigma (W_{anf,n3}^{T}\mathrm{ReLU}(W_{anf,n2}^{T}\mathrm{ReLU}(W_{anf,n1}^{T}h_{t})))\in [0,1]$$

where $h_{t}\in {\mathbb{R}}^{d}$ is the feature vector at the *t*-th time step and $W_{anf,n1}\in {\mathbb{R}}^{d\times d/2}$, $W_{anf,n2}\in {\mathbb{R}}^{d/2\times d/4}$, and $W_{anf,n3}\in {\mathbb{R}}^{d/4\times 1}$ are the weight matrices of the noise estimation network.

In parallel, the signal enhancement network generates enhanced features to amplify weak leak signal components:

(15) $$s_{t}=\tanh(W_{anf,s2}^{T}\mathrm{ReLU}(W_{anf,s1}^{T}h_{t}))\in {\left[-1,1\right]}^{d}$$

where $W_{anf,s1},W_{anf,s2}\in {\mathbb{R}}^{d\times d}$ are the weight matrices of the enhancement network. A gating network generates adaptive weights $g_{t}$ based on the original features and the estimated noise level:

(16) $$g_{t}=\sigma (W_{anf,g2}^{T}\mathrm{ReLU}(W_{anf,g1}^{T}[h_{t};n_{t}]))\in {\left[0,1\right]}^{d}$$

where $[h_{t};n_{t}]\in {\mathbb{R}}^{d+1}$ denotes the concatenation of the feature vector and the scalar noise level, and $W_{anf,g1}\in {\mathbb{R}}^{(d+1)\times d}$ and $W_{anf,g2}\in {\mathbb{R}}^{d\times d}$ are the gating network’s weight matrices.

The final filtered output is obtained through a three-term weighted fusion:

(17) $${\tilde{h}}_{t}=g_{t}⊙h_{t}+n_{t}s_{t}+(1-n_{t})h_{t}$$

The collection of all filtered vectors ${\tilde{h}}_{t}$ forms the final output matrix of the module, denoted as $H_{\mathrm{anf}}\in {\mathbb{R}}^{T\times d}$.

This design achieves noise-adaptive feature modulation: under high noise conditions ($n_{t}\to 1$), the model relies more on the enhanced signal $s_{t}$; under low noise conditions ($n_{t}\to 0$), the model primarily retains the original features while fine-tuning through the gating weights $g_{t}$. Compared to traditional frequency-domain filtering or wavelet denoising methods, this module can learn specialized noise suppression strategies for pipeline leak signals.

**Figure 4:**
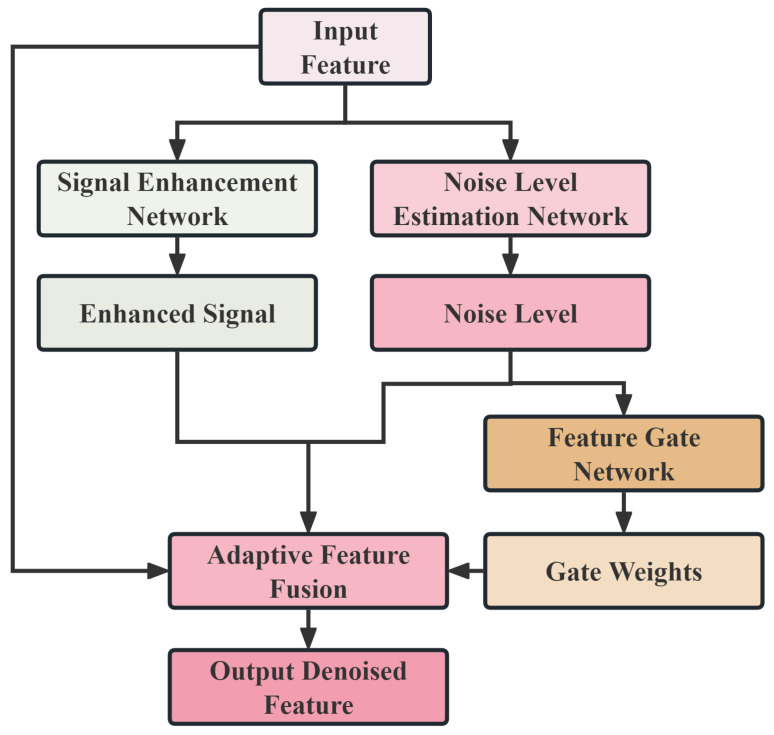
The architecture diagram of the adaptive noise filter.

The architecture of the adaptive noise filter is shown in Figure 4.

### 2.6. Overall Architecture and Training Objective

The MSFAT model adopts a hierarchical encoder-classifier architecture. The encoder part consists of L = 6 stacked encoder layers of identical structure, each sequentially comprising three sub-modules: multi-head frequency attention (MHFA), adaptive noise filtering (ANF), and a feed-forward network (FFN). To ensure stable training and efficient gradient propagation in deep networks, each sub-module employs residual connections and layer normalization:

(18) $$H^{\left(l\right)}=\mathrm{LayerNorm}(H^{\left(l-1\right)}+\mathrm{MHFA}(H^{\left(l-1\right)}))$$

(19) $${\tilde{H}}^{\left(l\right)}=\mathrm{LayerNorm}(\mathrm{ANF}(H^{\left(l\right)}))$$

(20) $$H^{\left(l\right)}=\mathrm{LayerNorm}({\tilde{H}}^{\left(l\right)}+\mathrm{FFN}({\tilde{H}}^{\left(l\right)}))$$

where l∈{1,2,…,L} denotes the layer index, and $H^{\left(0\right)}$ is the output of the frequency-aware embedding layer. The feed-forward network employs a standard two-layer MLP structure applied position-wise: $\mathrm{FFN}(X)=\mathrm{GELU}(XW_{ffn,1})W_{ffn,2}$, where $W_{ffn,1}\in {\mathbb{R}}^{d\times 4d}$ and $W_{ffn,2}\in {\mathbb{R}}^{4d\times d}$ are the learnable weight matrices.

The sequence features $H^{\left(L\right)}\in {\mathbb{R}}^{T\times d}$ output by the encoder need to be converted into a fixed-dimension global representation to support classification decisions. To fully leverage the sequence information, three complementary pooling strategies are employed:

(21) $$f_{avg}=\frac{1}{T}\sum_{t=1}^{T}h_{t}^{\left(L\right)}\in {\mathbb{R}}^{d}$$

(22) $$f_{max}=\max_{t=1}^{T}h_{t}^{\left(L\right)}\in {\mathbb{R}}^{d}$$

(23) $$f_{att}=\sum_{t=1}^{T}\alpha_{t}h_{t}^{\left(L\right)}\in {\mathbb{R}}^{d}$$

where $h_{t}^{\left(L\right)}$ is the feature vector at time step t from the final layer’s output $H^{\left(L\right)}$. The attention weights are computed as follows:

(24) $$\alpha_{t}=\frac{\exp(w_{pool}^{T}\mathrm{ReLU}(W_{pool}h_{t}^{\left(L\right)}))}{\sum_{j=1}^{T}\exp(w_{pool}^{T}\mathrm{ReLU}(W_{pool}h_{j}^{\left(L\right)}))}$$

Here, $W_{pool}\in {\mathbb{R}}^{d\times d/2}$ is a learnable weight matrix and $w_{pool}\in {\mathbb{R}}^{d/2}$ is a learnable weight vector for the attention mechanism. The three pooled features are concatenated and fed into the classification head:

(25) $$f_{global}=[f_{avg};f_{max};f_{att}]\in {\mathbb{R}}^{3d}$$

The classifier then uses a three-layer MLP with Dropout to produce the final probability distribution p:

(26) $$z_{1}=\mathrm{ReLU}(f_{global}^{T}W_{\mathrm{cls},1})$$

(27) $$z_{2}=\mathrm{ReLU}(\mathrm{Dropout}(z_{1})W_{\mathrm{cls},2})$$

(28) $$p=\mathrm{Softmax}(\mathrm{Dropout}(z_{2})W_{\mathrm{cls},3})\in {\mathbb{R}}^{3}$$

where $W_{\mathrm{cls},1}\in {\mathbb{R}}^{3d\times d}$, $W_{\mathrm{cls},2}\in {\mathbb{R}}^{d\times d/2}$, and $W_{\mathrm{cls},3}\in {\mathbb{R}}^{d/2\times 3}$ are the weight matrices of the classifier.

The model is trained end-to-end using the cross-entropy loss:

(29) $$L=-\frac{1}{N}\sum_{i=1}^{N}\sum_{c=0}^{2}y_{i}^{\left(c\right)}\log p_{i}^{\left(c\right)}$$

where *N* is the batch size, $y_{i}^{\left(c\right)}$ is the one-hot encoded true label for the *i*-th sample, and $p_{i}^{\left(c\right)}$ is the corresponding predicted probability.

## 3. Experimental Setup

### 3.1. Dataset Description

The experimental dataset employed in this study is derived from the publicly published research study by Meng et al. [60] and is openly accessible on GitHub (https://github.com/mengdinet/Gas-pipeline-leakage-data-set, accessed on 1 August 2025). for use by the scientific community. The dataset focuses on acoustic signal-based gas pipeline leak detection. Data acquisition was based on a high-fidelity, professional-grade experimental measurement system designed to ensure metrological rigor and industrial relevance. The system consisted of a physical pipeline network with a main pipe diameter of 125 mm and a branch pipe diameter of 25 mm. To accurately simulate early micro-leakages commonly encountered in industrial settings, researchers constructed two typical leakage scenarios by machining a 0.2 mm diameter hole in the pipeline gasket and controlling valve operations.

Acoustic signals were acquired non-contractively by a high-sensitivity 32-channel helical microelectromechanical system (MEMS) microphone array positioned 6 m from the leak source and digitized through a field-programmable gate array (FPGA) at a sampling rate of up to 96 kHz. This professional instrumentation is crucial for ensuring the comprehensive capture of high-frequency leakage characteristics and providing high-fidelity raw data for subsequent analysis, offering a level of precision unattainable by consumer-grade multimedia devices.

To process the multi-channel recordings, a definitive channel selection strategy was adopted. This study focuses on single-channel signal analysis; therefore, no multi-channel fusion methods were applied. For each measurement, the single channel exhibiting the highest signal-to-noise ratio (SNR) was selected from the 32-channel data to serve as the input for our model. This standard pre-processing step ensures that the model is supplied with a signal of the highest possible quality, allowing it to focus on the intrinsic time-frequency characteristics of the acoustic events.

To ensure the engineering application value and metrological rigor of the dataset, key parameters during data acquisition were strictly controlled. Gas pressure within the pipeline was precisely regulated between 0.4 MPa and 0.8 MPa, strictly adhering to the Chinese national standard “Code for Design of City Gas Engineering” (GB 50028-2006) [61], providing clear standard traceability and engineering validity for experimental conditions. Furthermore, to simulate noise interference in real industrial environments, the experiment synthesized collected background fan noise with pure leakage signals, systematically generating samples with SNR varying between 5 dB and 30 dB. This quantitative control of SNR enables the dataset to effectively evaluate algorithm robustness under different quantified noise levels, which is crucial for assessing the performance limits of detection systems.

Finally, the original audio with a total duration of 6.25 h was processed into 22,500 independent samples, each with a duration of 1 s. Each sample was explicitly labeled as one of three categories: “no-leakage” (pure background noise), “hole-leakage” (0.2 mm hole leakage mixed with noise), and “valve-leakage” (valve leakage mixed with noise). To prevent data leakage, dataset partitioning is performed by grouping based on acquisition conditions. The original audio forms multiple independent acquisition groups according to different combinations of experimental pressure, leakage type, and noise level. Partitioning first allocates acquisition groups to training and test sets at an 80/20 ratio, then assigns all 1-s samples within each group entirely to the corresponding subset, ensuring samples from the same acquisition group never appear across sets. This strategy effectively avoids information leakage between adjacent temporal windows. The final training set contains 18,000 samples, while the test set contains 4500 samples. All performance evaluations employ 5-fold cross-validation with data re-partitioning by acquisition groups in each fold, ensuring that test sets always contain independent acquisition groups unseen during training.

### 3.2. Evaluation Metrics

This study employs accuracy and F1-score as the primary evaluation metrics to comprehensively assess the classification performance of the MSFAT model in pipeline leak detection tasks.

Accuracy is defined as the ratio of correctly classified samples to the total number of samples:

(30) $$\mathrm{Accuracy}=\frac{TP+TN}{TP+TN+FP+FN}$$

where *TP*, *TN*, *FP*, and *FN* represent the numbers of true positives, true negatives, false positives, and false negatives, respectively.

The F1-score is the harmonic mean of precision and recall, calculated as follows:

(31) $$\mathrm{Precision}=\frac{TP}{TP+FP}$$

(32) $$\mathrm{Recall}=\frac{TP}{TP+FN}$$

(33) $$F1-\mathrm{score}=\frac{2\times \mathrm{Precision}\times \mathrm{Recall}}{\mathrm{Precision}+\mathrm{Recall}}$$

For multi-class tasks, the macro-averaged F1-score is adopted as the comprehensive evaluation metric:

(34) $$\mathrm{Macro}\;F1=\frac{1}{C}\sum_{c=1}^{C}{F1}_{c}$$

where *C* = 3 is the number of classes and ${F1}_{c}$ is the F1-score for the *c*-th class.

### 3.3. Implementation Details

The training of MSFAT is implemented using the PyTorch deep learning framework, with all experiments conducted on a workstation equipped with an NVIDIA RTX 4090 GPU. The key hyperparameter configuration of MSFAT is shown in Table 1. All experiments employ 5-fold cross-validation with data re-partitioning by acquisition groups in each fold. Reported metrics are 5-fold averages with confidence intervals calculated via t-distribution. This strategy validates generalization to unseen acquisition conditions, ensuring unbiased results.

## 4. Experimental Results and Discussions

### 4.1. Parameter Analysis

To gain a comprehensive understanding of the relationship between the performance of the MSFAT model and its key hyperparameters, this section conducts systematic sensitivity analysis on the number of encoder layers and the number of attention heads in the multi-head attention mechanism. These two hyperparameters directly affect the model’s representation learning capability and computational complexity, and their rational configuration is crucial for achieving optimal performance in pipeline leak detection tasks.

**Figure 5:**
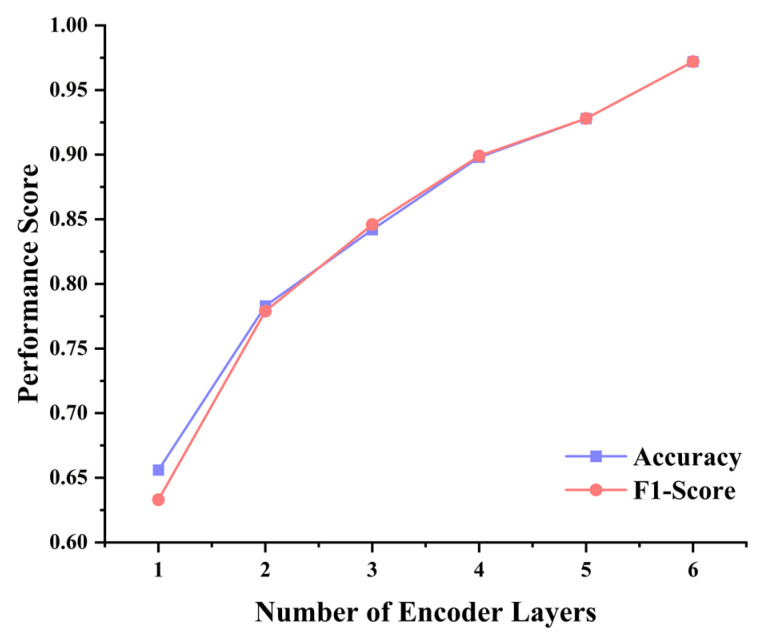
Performance analysis of different numbers of encoder layers.

The number of encoder layers determines the depth and feature abstraction capability of the MSFAT model. As shown in Figure 5, MSFAT performance exhibits a significant monotonic increasing trend with the increase in the number of encoder layers. When the number of layers increases from 1 to 6, accuracy improves from 0.656 to 0.972, and the F1-score increases from 0.633 to 0.972, with improvement margins of 0.316 and 0.339 for accuracy and F1-score, respectively.

It is particularly noteworthy that when the number of layers increases from 5 to 6, accuracy still shows a significant improvement of 0.044, and the F1-score also demonstrates a corresponding increase of 0.044, indicating that deep network structures possess important value for modeling complex time-frequency patterns in acoustic signals. From the trend of F1-score changes, accuracy and F1-score maintain high consistency across different layer configurations, suggesting that MSFAT achieves relatively balanced classification performance across different categories without obvious class bias issues. This result validates the effectiveness of the MSFAT architecture design, demonstrating that the deep frequency-aware attention mechanism and adaptive noise filtering module can progressively extract more abstract and discriminative feature representations layer by layer.

**Figure 6:**
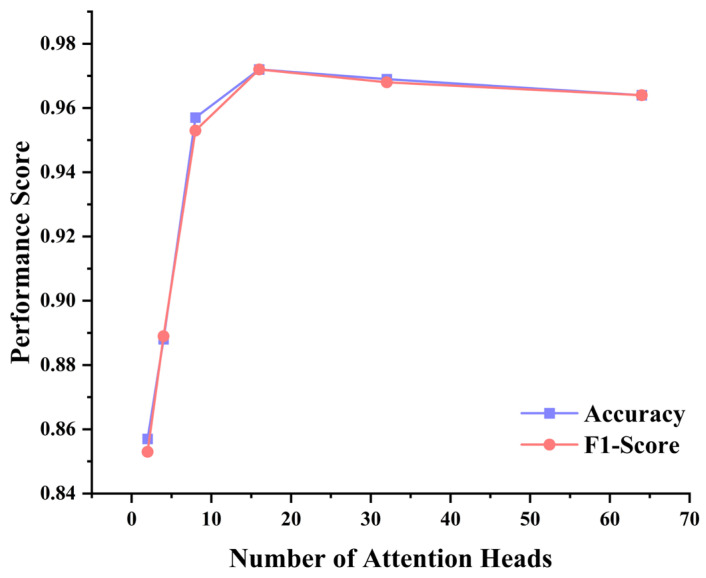
Performance analysis of different numbers of attention heads.

Building upon this foundation, the number of attention heads in the multi-head attention mechanism directly affects the model’s parallel processing capability for different frequency components. As shown in Figure 6, the number of attention heads has a significant impact on MSFAT performance but exhibits a non-monotonic change trend. When the number of attention heads increases from 2 to 16, accuracy steadily improves from 0.857 to 0.972, and the F1-score increases from 0.853 to 0.972, with improvement margins of 0.115 and 0.119 for accuracy and F1-score, respectively.

However, when the number of attention heads exceeds 16, model performance begins to show slight degradation. Under the 32-head configuration, accuracy decreases to 0.969 and the F1-score decreases to 0.968; under the 64-head configuration, accuracy further decreases to 0.964 and the F1-score decreases to 0.964. This phenomenon can be attributed to increased model complexity and potential overfitting risks caused by excessive parameterization. From the change pattern of the F1-score, it exhibits a similar trend to accuracy, but the decline magnitude of the F1-score is slightly larger than that of accuracy under high attention head configurations, which may indicate that excessive attention heads have a more pronounced negative impact on the model’s balanced classification capability across different categories. Additionally, excessive attention heads may lead to redundant computations in the frequency-aware attention mechanism, weakening the functional differentiation between different heads.

Comprehensive analysis indicates that the configuration of 6 encoder layers and 16 attention heads represents the optimal hyperparameter combination for MSFAT in pipeline leak detection tasks, achieving both accuracy and an F1-score of 0.972 while maintaining reasonable computational complexity.

### 4.2. Comparative Analysis

To comprehensively evaluate the performance advantages of the MSFAT model in pipeline leak detection tasks, this section conducts comparative analysis with various mainstream deep learning architectures. To comprehensively evaluate the performance advantages of the MSFAT model in pipeline leak detection tasks, this section conducts comparative analysis with various mainstream deep learning architectures. Before presenting the quantitative results, it is crucial to clarify the methodological novelty of our approach in relation to the work of Meng et al. [60], who provided the public dataset for this study. While the contribution of Meng et al. was the development of an efficient Convolutional Neural Network (CNN) architecture, our research introduces a fundamentally different paradigm based on a Multi-Scale Frequency-Aware Transformer (MSFAT). The core innovation of MSFAT lies not in the data but in its architecture, which deeply integrates domain-specific knowledge of acoustic signals. This is achieved through key components absent in traditional CNNs, such as the frequency-aware embedding layer for joint time-frequency analysis, the multi-head frequency attention mechanism guided by spectral distribution, and the adaptive noise filtering module. Therefore, the following performance comparison serves not only to demonstrate MSFAT’s superior metrics but also to validate the effectiveness of this novel, frequency-aware Transformer architecture against established baselines.

As shown in Table 2, MSFAT achieves optimal performance among all compared models, with both accuracy and F1-score reaching 0.972 and a confidence interval of [0.968, 0.976]. All metrics are based on 5-fold cross-validation with confidence intervals calculated via t-distribution. McNemar’s test demonstrates MSFAT exhibits statistically significant advantages over all baseline models (*p* < 0.001), confirming performance improvements stem from architectural innovations rather than random fluctuations. Statistical significance was assessed using McNemar’s test, adapted for the multi-class cross-validation framework. For each pairwise comparison between MSFAT and a baseline model, the prediction outcome for every test sample across all five folds was first binarized to either correct or incorrect. The test’s logic is centered on analyzing the asymmetry of disagreements between the models, with its test statistic being calculated from the aggregated counts that form a 2 × 2 contingency table.

In contrast, the standard Transformer model, which serves as our primary baseline, achieves a significantly lower accuracy of 0.867, an F1-score of 0.863, and a confidence interval of [0.861, 0.873]. This results in a substantial performance improvement of 10.5% in accuracy and 10.9% in F1-score for MSFAT. This significant gain is primarily attributed to our novel architectural designs that address the inherent limitations of the standard Transformer in processing acoustic signals. Specifically, the frequency-aware embedding layer allows the model to capture critical time-frequency dual-domain features, which are often missed by standard single-domain processing. Furthermore, the multi-head frequency attention mechanism enables the model to utilize frequency-domain prior knowledge to focus on leak-related spectral features, a capability the standard self-attention mechanism lacks. Finally, the adaptive noise filtering module enhances robustness in noisy environments. These integrated innovations collectively validate the effectiveness and superiority of our proposed approach.

Regarding convolutional neural networks, ResNet18, as a classic deep residual network that performs excellently in computer vision, achieves only 0.914 accuracy and a 0.913 F1-score in acoustic signal processing tasks, which are 0.058 and 0.059 lower than MSFAT, respectively. This result indicates that although traditional convolutional architectures can extract local features, they lack specialized modeling capabilities for the time-frequency characteristics of acoustic signals and struggle to fully utilize frequency-domain prior knowledge in pipeline leak signals.

In contrast, models specifically designed for audio tasks demonstrate stronger adaptability. AST applies the Transformer architecture to patch sequences of mel spectrograms, with its global self-attention mechanism capable of capturing long-range dependencies across both time and frequency dimensions in spectrograms, achieving an accuracy of 0.925 and an F1-score of 0.931, reflecting the effectiveness of self-attention in spectral feature aggregation. RCNN employs convolutional layers to extract local time-frequency patterns before utilizing recurrent layers to model temporal evolution characteristics, achieving an accuracy of 0.916 and an F1-score of 0.916, demonstrating the complementary advantages of hybrid architectures in processing acoustic signals. PANN, pretrained on the large-scale AudioSet dataset, learns general audio representation capabilities, achieving an accuracy of 0.911 and an F1-score of 0.909, though its generality results in insufficient specificity toward the particular frequency patterns of pipeline leaks. While these models already possess considerable capability in audio processing, MSFAT achieves further performance improvement in the specific task of pipeline leak detection through targeted designs such as frequency-aware embedding and adaptive noise filtering.

In the recurrent neural network category, bidirectional models generally outperform unidirectional models. BiGRU achieves an accuracy of 0.856 and an F1-score of 0.856, showing clear improvements compared to the unidirectional GRU’s 0.833 and 0.834. BiLSTM achieves an accuracy of 0.813 and an F1-score of 0.815, similarly outperforming LSTM’s 0.757 and 0.753. However, even the best-performing BiGRU still significantly lags behind MSFAT, with accuracy and F1-score being 0.116 and 0.116 lower, respectively. This indicates that traditional recurrent architectures suffer from gradient vanishing and information loss problems when processing long-sequence acoustic signals, making it difficult to effectively model long-range temporal dependencies.

It is noteworthy that among all compared models, accuracy and F1-score maintain high consistency, with differences within 0.004, indicating minor class bias issues. MSFAT’s perfect consistency between the two metrics further demonstrates its balanced cross-category classification capability. Confidence interval analysis reveals the correlation between model performance and prediction stability: AST [0.920, 0.930] and RCNN [0.911, 0.921] exhibit narrow intervals corresponding to high accuracy, while LSTM [0.749, 0.765] shows a wider interval reflecting insufficient prediction consistency. MSFAT’s narrowest confidence interval confirms its superior generalization stability.

**Figure 7:**
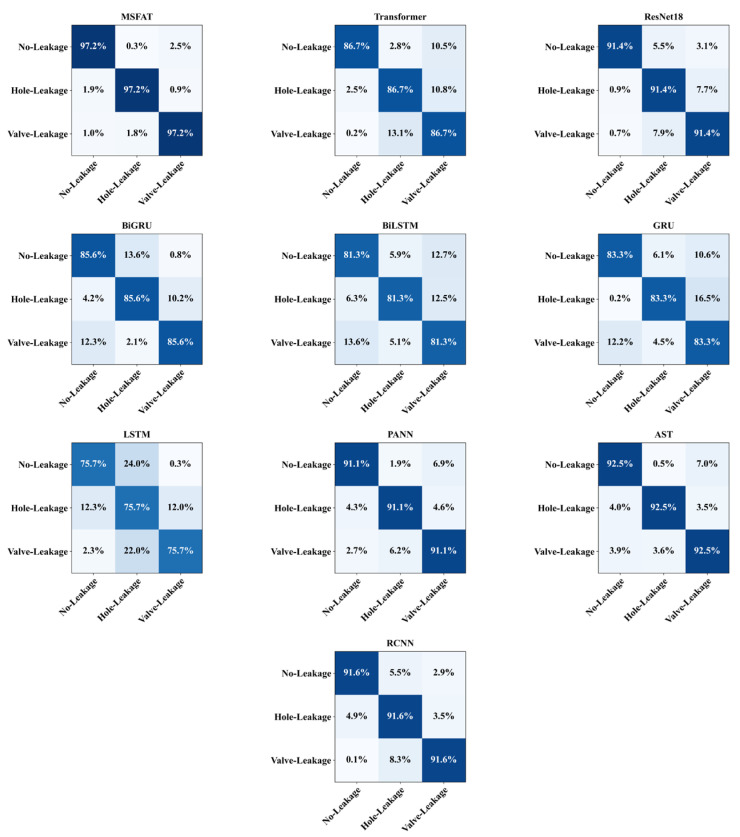
The confusion matrices of baseline models and MSFAT.

Confusion matrix analysis in Figure 7 reveals classification performance differences across models. MSFAT exhibits minimal inter-class confusion across all three leakage categories with highly symmetric misclassification distributions, demonstrating exceptional class discrimination capability. Traditional recurrent networks like LSTM show severe confusion across all categories, while Transformer exhibits notable misclassification in valve-leakage recognition. In contrast, audio-specialized models AST and PANN demonstrate better inter-class separation, yet still fall short of MSFAT’s balanced performance. Confusion matrix results further confirm that frequency-aware mechanisms effectively suppress inter-class confusion.

Comprehensive comparison results demonstrate that MSFAT achieves significant performance improvements in pipeline leak detection tasks compared to traditional deep learning methods through the integration of frequency-aware mechanisms and adaptive noise processing capabilities, validating the necessity and effectiveness of specialized design for acoustic signal characteristics.

### 4.3. SNR Robustness Analysis

Pipeline leak detection systems in industrial environments must maintain stable detection performance under complex and variable noise conditions. To evaluate the robustness of the MSFAT model under different signal-to-noise ratio conditions, this section systematically analyzes the model’s performance under SNR conditions ranging from 5 dB to 30 dB. This experiment simulates noise interference in real industrial environments by mixing collected background noise with pure leak signals at different ratios.

As shown in Table 3, the MSFAT model demonstrates robust detection performance under all tested SNR conditions, as measured by both accuracy and F1-Score. Under the most severe 5 dB SNR condition, the model achieves an impressive 0.875 in both metrics. As the SNR improves, both metrics show a stable monotonic increasing trend. When the SNR increases from 5 dB to 30 dB, accuracy grows from 0.875 to 0.952, while the F1-Score follows a similarly strong monotonic trend, rising from 0.875 to 0.952. The close alignment between these two metrics is particularly noteworthy, as it indicates that the model maintains balanced classification performance across all classes even under significant noise interference, thereby confirming its stability and lack of significant class bias.

It is particularly noteworthy that even under extremely low SNR (5 dB) conditions, MSFAT’s detection accuracy remains at the relatively high level of 0.875, which fully demonstrates the effectiveness of the adaptive noise filtering module. This module can effectively extract weak leak characteristic signals under strong noise interference through end-to-end noise detection, signal enhancement, and adaptive fusion mechanisms. From the performance growth curve, within the moderate SNR range (10–20 dB), model performance improvement is relatively gradual, with each 5 dB SNR improvement bringing approximately 0.012–0.020 accuracy enhancement, while within the high SNR range (20–30 dB), performance improvement is more significant, with each 5 dB improvement bringing approximately 0.021–0.015 accuracy growth.

This robustness performance can be attributed to the synergistic effects of multiple key components in the MSFAT architecture. The frequency-aware embedding layer maintains sensitivity to leak-related frequency bands under noise interference through time-frequency dual-domain feature fusion; the multi-head frequency attention mechanism dynamically adjusts attention weights according to spectral distribution, effectively suppressing interference from noise frequency bands; and the adaptive noise filtering module implements adaptive processing strategies for different noise levels.

Comprehensive analysis indicates that the MSFAT model can maintain stable detection performance under wide-range SNR conditions, validating its practicality and reliability in complex industrial environments. This robustness advantage enables MSFAT to adapt to pipeline leak detection requirements under different operating conditions, providing an important technical guarantee for practical engineering applications.

### 4.4. Limited Data Performance Analysis

To evaluate the MSFAT model’s learning capability and generalization performance under data-scarce conditions, this section analyzes the model’s detection performance across different training data ratios. The experiment simulates challenging scenarios in practical engineering applications by progressively reducing training data volume. This approach validates the model’s practicality when annotated data are limited.

**Figure 8:**
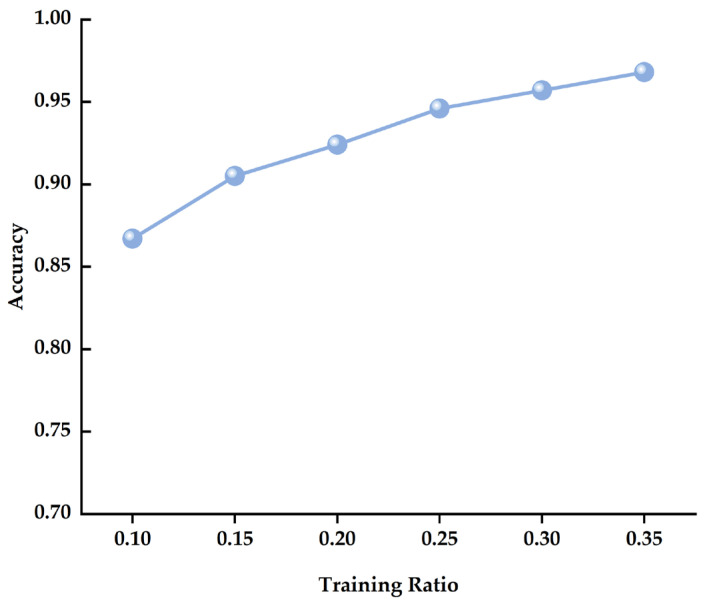
Performance analysis under limited data conditions.

Figure 8 reveals that the MSFAT model maintains strong learning capability under limited data conditions. The model achieves 0.867 accuracy with only 10% training data. Performance shows a stable upward trend as the training data ratio increases. Accuracy improves from 0.867 to 0.968 when training data grows from 10% to 35%. This represents a performance gain of 0.101.

Notably, MSFAT maintains relatively high detection accuracy even with extremely limited training data at 10%. This superior performance stems from two key factors. The frequency-aware embedding layer provides efficient representation learning for acoustic signal time-frequency features. Meanwhile, the multi-head frequency attention mechanism effectively utilizes frequency-domain prior knowledge.

The performance growth curve analysis reveals distinct patterns across different data ratio ranges. In the low data range from 10% to 20%, each 5% increase in training data yields approximately a 0.019 accuracy improvement. Performance improvement becomes more pronounced in the moderate range from 20% to 30%. Here, each 5% data increase brings roughly 0.022 accuracy growth. However, performance growth plateaus in the high data range from 30% to 35%. Only 0.011 improvement occurs in this range. This indicates the model approaches its performance ceiling on the current dataset.

These results demonstrate that the MSFAT architecture possesses excellent data efficiency. The model achieves effective pipeline leak detection under limited annotated data conditions. This provides a viable technical solution for data scarcity challenges in practical engineering applications.

### 4.5. Ablation Study

To thoroughly validate the effectiveness and necessity of each innovative module in the MSFAT architecture, this section designs systematic ablation experiments. By progressively removing or replacing key components, we quantitatively analyze each module’s contribution to overall performance, providing empirical support for the rationality of the model design. The ablation experiments cover core components, including the frequency-aware embedding layer, multi-head frequency attention mechanism, adaptive noise filtering module, and multi-scale feature aggregation strategy.

As shown in Table 4, the complete MSFAT model achieves optimal performance with both accuracy and F1-score reaching 0.972. When the frequency-aware embedding layer is removed (w/o FAE) and standard single-domain embedding is adopted, model performance significantly decreases, with accuracy dropping to 0.918 and F1-score to 0.914, representing performance losses of 0.054 and 0.058, respectively. This result indicates that joint representation learning of time-frequency dual-domain features plays a crucial role in acoustic signal analysis, and relying solely on time-domain or frequency-domain features cannot adequately capture the complex patterns of pipeline leak signals.

The removal of the multi-head frequency attention mechanism (w/o MHFA) leads to the most severe performance degradation, with accuracy and F1-score dropping to 0.896 and 0.892, respectively, representing performance losses of 0.076 and 0.080 relative to the complete model. This phenomenon demonstrates the critical importance of frequency-aware attention computation for pipeline leak detection tasks. Although standard multi-head self-attention mechanisms can model dependencies within sequences, they lack effective utilization of frequency-domain prior knowledge and struggle to accurately identify leak-related spectral features.

The absence of the adaptive noise filtering module (w/o ANF) causes model accuracy to drop to 0.931 and the F1-score to 0.928, with performance losses of 0.041 and 0.044, respectively. This indicates that the adaptive noise filtering module plays an important signal enhancement role in complex acoustic environments, with its end-to-end noise suppression strategy demonstrating obvious advantages over traditional pre-processing methods.

The removal of the multi-scale feature aggregation strategy (w/o MSA) leads to accuracy and F1-score dropping to 0.943 and 0.940, respectively, with performance losses of 0.029 and 0.032. This result proves the complementary value of multiple pooling strategies. Although single global average pooling can provide basic sequence representation, it cannot fully utilize the saliency information and attention-weighted features in sequences.

To further analyze the synergistic effects between modules, the experiment also tests the joint removal effects of multiple modules. When both the frequency-aware embedding layer and multi-head frequency attention mechanism are simultaneously removed (w/o FAE + MHFA), model performance drops drastically, with accuracy and F1-score falling to 0.847 and 0.841, respectively, representing performance losses of 0.125 and 0.131. This result indicates significant synergistic effects between the two frequency-aware modules, with their joint effect exceeding the simple addition of their individual contributions.

In contrast, the simultaneous removal of the adaptive noise filtering module and multi-scale feature aggregation strategy (w/o ANF + MSA) results in relatively smaller performance losses, with accuracy and F1-score dropping to 0.908 and 0.904, respectively, representing losses of 0.064 and 0.068. This further validates the core position of frequency-aware mechanisms in the MSFAT architecture.

From a technical mechanism perspective, the frequency-aware embedding layer provides a rich representational foundation for subsequent processing through time-frequency dual-domain feature fusion, while the multi-head frequency attention mechanism dynamically adjusts attention distribution based on spectral priors. Together, these two components constitute the core advantages of MSFAT in processing acoustic signals. Although the adaptive noise filtering module and multi-scale feature aggregation strategy contribute relatively less, they play important roles in enhancing model robustness and representational completeness.

Comprehensive ablation experiment results demonstrate that each innovative component in the MSFAT architecture contributes positively to the final performance, with frequency-aware related modules being most critical, validating the necessity and effectiveness of specialized design for acoustic signal characteristics.

### 4.6. Computational Efficiency Analysis

Pipeline leak detection systems in industrial environments require not only high-precision classification performance but also real-time response capabilities and large-scale cloud deployment requirements. To evaluate the engineering practicality of the MSFAT model, this section systematically analyzes key performance indicators, including computational complexity, inference efficiency, and memory footprint.

Table 5 presents the computational performance metrics of the MSFAT model. All performance metrics, including inference time and computational load (FLOPs), were benchmarked on a workstation equipped with an NVIDIA RTX 4090 GPU and an Intel Core i9-14900HX CPU, using PyTorch 2.1 and CUDA 12.1. The model contains 30.45 M total parameters, 26.964 G FLOPs, an average inference time of 31.78 ± 1.48 ms, model memory of 121.16 MB, and inference memory of 130.16 MB.

Comprehensive performance metrics demonstrate that MSFAT achieves a favorable balance between computational efficiency and model complexity while ensuring high classification accuracy. The inference time of 31.78 ms with a low standard deviation of 1.48 ms ensures a stable real-time response, while the memory footprint of 121.16 MB enables a single server to load dozens of model instances in parallel, supporting centralized monitoring of large-scale distributed pipeline networks. Compared to large-scale pretrained models, MSFAT’s lightweight design significantly enhances cloud server utilization efficiency and reduces deployment costs. Its compact architecture simplifies version updates and distributed deployment procedures, providing a technical guarantee for unified management of cross-regional pipeline networks and validating the feasibility of large-scale deployment in cloud-based industrial monitoring systems.

## 5. Conclusions

This study addresses key issues in pipeline leakage detection, including insufficient utilization of time-frequency domain features, poor adaptability to noisy environments, and inadequate utilization of frequency-domain prior knowledge by proposing the Multi-Scale Frequency-Aware Transformer (MSFAT) architecture. Through the frequency-aware embedding layer that achieves joint representation learning of time-frequency dual-domain features, the multi-head frequency attention mechanism that dynamically adjusts attention weights according to spectral distribution, and the adaptive noise filtering module that realizes end-to-end joint optimization, the approach effectively overcomes the technical limitations of traditional methods. Experimental results demonstrate that MSFAT achieves 0.972 in both accuracy and F1-score. This marks a significant improvement of 10.5% and 10.9%, respectively, when compared to a standard Transformer baseline. This notable enhancement is not merely incremental; it stems directly from the deep integration of acoustic signal properties into the model architecture. Unlike the standard Transformer, which is agnostic to signal characteristics, MSFAT’s frequency-aware mechanisms (including the embedding layer and attention mechanism) empower it to precisely identify and leverage leak-specific spectral patterns. As confirmed by our ablation studies, these frequency-aware components are the most significant contributors to the performance gain. Furthermore, the model’s robustness across a 5–30 dB signal-to-noise ratio range validates the effectiveness of the adaptive noise filtering module in complex industrial environments. The theoretical contribution of this research lies in deeply integrating the physical properties of acoustic signals into deep learning architecture design, providing a new technological paradigm for the field of acoustic signal analysis; the practical value is reflected in providing a high-precision, robust intelligent detection solution for industrial pipeline safety monitoring. However, the research is based solely on datasets from specific experimental environments, and future study needs to validate the model’s generalization capability in more diverse industrial scenarios, explore the potential of multi-channel fusion techniques such as late fusion or cross-channel attention to further boost performance, and explore lightweight deployment strategies to meet real-time detection requirements.

## Figures and Tables

**Table 1:** Hyperparameter configuration of the MSFAT model.

Parameter	Value
Embedding dimension (d)	512
Number of encoder layers (L)	6
Number of attention heads (h)	16
FFN hidden dimension	2048
Dropout rate	0.1
Learning rate	1 × 10^−4^
Batch size	32
Training epochs	100
Optimizer	Adam

**Table 2:** Performance comparison with baseline models (5-fold cross-validation).

Model	Accuracy	95% CI	F1-Score	Significance
MSFAT	0.972	[0.968, 0.976]	0.972	-
Transformer	0.867	[0.861, 0.873]	0.863	*p* < 0.001
ResNet18	0.914	[0.909, 0.919]	0.913	*p* < 0.001
BiGRU	0.856	[0.850, 0.862]	0.856	*p* < 0.001
BiLSTM	0.813	[0.806, 0.820]	0.815	*p* < 0.001
GRU	0.833	[0.827, 0.839]	0.834	*p* < 0.001
LSTM	0.757	[0.749, 0.765]	0.753	*p* < 0.001
PANN	0.911	[0.906, 0.916]	0.909	*p* < 0.001
AST	0.925	[0.920, 0.930]	0.931	*p* < 0.001
RCNN	0.916	[0.911, 0.921]	0.916	*p* < 0.001

**Table 3:** Performance analysis under different SNR conditions.

SNR (dB)	5	10	15	20	25	30
Accuracy	0.875	0.884	0.904	0.916	0.937	0.952
F1-Score	0.875	0.882	0.903	0.915	0.936	0.952

**Table 4:** Ablation study results.

Configuration	Accuracy	F1-Score
**MSFAT (Full)**	**0.972**	**0.972**
w/o FAE	0.918	0.914
w/o MHFA	0.896	0.892
w/o ANF	0.931	0.928
w/o MSA	0.943	0.940
w/o FAE + MHFA	0.847	0.841
w/o ANF + MSA	0.908	0.904

**Table 5:** Computational performance metrics of the MSFAT model.

Metric	Value
Total parameters	30.45 M
FLOPs	26.964 G
GPU inference time	31.78 ± 1.48 ms
Model memory	121.16 MB
Inference memory	130.16 MB

## Data Availability

The experimental dataset used in this study, created by Meng et al. [60], is openly accessible on GitHub (https://github.com/mengdinet/Gas-pipeline-leakage-data-set, accessed on 1 August 2025). The data preparation, designed to ensure evaluation independence, involved these key steps: (1) Channel Selection: From the original 32-channel recordings, the single channel with the highest Signal-to-Noise Ratio (SNR) was selected for each measurement. (2) Data Partitioning: To prevent data leakage, partitioning was performed at the “acquisition group” level, ensuring that samples from the same acquisition event do not appear across the training set (18,000 samples) and the test set (4500 samples). This process is described in detail in Section 3.1 of this paper. To ensure full reproducibility, the source code for the proposed MSFAT model will be made publicly available on GitHub upon publication of this article.

## Abbreviations

The following abbreviations are used in this manuscript:

MSFAT	Multi-Scale Frequency-Aware Transformer
SNR	signal-to-noise ratio
RFFT	real-valued fast Fourier transform
MHFA	multi-head frequency attention
ANF	adaptive noise filtering
FFN	feed-forward network
MEMS	microelectromechanical system
FPGA	field-programmable gate array
